# Baseline Complete Blood Count and Chemistry Panel Profile from the Japan Environment and Children’s Study (JECS)

**DOI:** 10.3390/ijerph19063277

**Published:** 2022-03-10

**Authors:** Yu Taniguchi, Shin Yamazaki, Shoji F. Nakayama, Makiko Sekiyama, Takehiro Michikawa, Tomohiko Isobe, Miyuki Iwai-Shimada, Yayoi Kobayashi, Mai Takagi, Michihiro Kamijima, The Japan Environment and Children’s Study Group

**Affiliations:** 1 Japan Environment and Children’s Study Programme Office, National Institute for Environmental Studies, Tsukuba 305-8506, Japan; taniguchi.yu@nies.go.jp (Y.T.); fabre@nies.go.jp (S.F.N.); sekiyama.makiko@nies.go.jp (M.S.); isobe.tomohiko@nies.go.jp (T.I.); iwai.miyuki@nies.go.jp (M.I.-S.); kobayashi.yayoi@nies.go.jp (Y.K.); takagi.mai@nies.go.jp (M.T.); 2 Department of Environmental and Occupational Health, Toho University School of Medicine, Ota 143-8540, Japan; takehiro.michikawa@med.toho-u.ac.jp; 3 Department of Occupational and Environmental Health, Nagoya City University Graduate School of Medical Sciences, Nagoya 467-8601, Japan; kamijima@med.nagoya-cu.ac.jp

**Keywords:** birth cohort, epidemiology, blood cell count, chemistry

## Abstract

Background: The Japan Environment and Children’s Study (JECS) is a nationwide birth cohort study of environmental factors affecting children’s health and development. We summarize the complete blood count and chemistry panel statistical data from pregnant women enrolled in JECS. Methods: Statistical data of up to 58,056 mother’s sample in their first (gestational age ≤ 13 weeks) and second trimester (22–27 weeks) were calculated. Results: Mean (SD) values in the first trimester were: white blood cell count, 7866 (1906)/μL; red blood cell count, 417 (33) 10^4^/μL; hemoglobin, 12.4 (1.0) g/dL; hematocrit, 37.1 (2.6)%; mean corpuscular volume, 89.2 (4.7) fL; mean corpuscular hemoglobin, 29.8 (1.9) pg; mean corpuscular hemoglobin concentration, 33.3 (0.9)%; platelet count, 24.8 (5.2) 10^4^/μL; HbA1c, 5.26 (0.26)%; total cholesterol, 181 (28) mg/dL; low density lipoprotein cholesterol, 95 (23) mg/dL; high density lipoprotein cholesterol, 73 (13) mg/dL; free cholesterol, 41 (7) mg/dL; triglycerides, 109 (47) mg/dL; total protein, 6.9 (0.4) g/dL; and albumin, 4.1 (0.2) g/dL. Mean values (SD) in the second trimester were: total cholesterol, 246 (38) mg/dL; free cholesterol, 61 (9) mg/dL; triglycerides, 183 (70) mg/dL; total protein, 6.5 (0.4) g/dL; and albumin, 3.6 (0.2) g/dL. Conclusions: These data will be useful for future JECS studies.

## 1. Introduction

The Japan Environment and Children’s Study (JECS) is a nationwide birth cohort study launched in January 2011 to evaluate the effects of chemical exposure during the fetal stage and in early childhood on children’s health and development [1]. Recruitment of women in early pregnancy was conducted from launch until March 2014, during which a total of 103,099 pregnancies were registered [2]. Based on selected maternal and infant characteristics in the first year of recruitment, the JECS cohort is representative of the general Japanese population [3]. Details of the JECS concept and design have been published elsewhere [1,2].

JECS examines the effects of exposure to a wide range of environmental factors [1] using questionnaires [3,4,5,6,7], chemical analysis of bio-specimens [8,9,10,11], environmental measurements [12,13], and atmospheric simulation based on monitoring of ambient air quality. Previous studies in JECS have reported various profile data on chemical analyses of bio-specimens, including the allergic profiles of mothers and fathers [8], metabolic status in pregnant women [9], concentrations of metallic elements in pregnant women [10], and urinary cotinine in pregnant women [11]. 

To our knowledge, no previous study has reported the complete blood count and chemistry panel data of a large sample of pregnant women in Japan. Further, there are no standard blood data values. In this descriptive study, we summarized the complete blood count and chemistry panel data of 58,056 mothers in their first and second trimesters with singleton pregnancies and live births. To examine regional differences in complete blood count and chemistry panel data in Japan, we also summarized the values for six regions. We hope that the complete blood count and chemistry panel data provided in this study will facilitate the establishment of standard values for Japanese pregnant women. The data from this study will also be useful for future studies in JECS that aim to examine the effect of maternal biochemistry on children’s health and development. 

## 2. Materials and Methods

### 2.1. Study Participants

JECS is funded by Japan’s Ministry of the Environment. The ultimate goal of JECS is “to identify environmental factors that affect children’s health and development in order to help decision-makers design better chemical risk management strategies” [1]. JECS involves collaborations between the Program Office (National Institute for Environmental Studies), the Medical Support Center (National Centre for Child Health and Development), and 15 Regional Centers (Hokkaido, Miyagi, Fukushima, Chiba, Kanagawa, Koshin, Toyama, Aichi, Kyoto, Osaka, Hyogo, Tottori, Kochi, Fukuoka, and South Kyushu/Okinawa) [2]. A total of 103,099 pregnancies were recruited and registered at the 15 Regional Centers across Japan via Co-operating health care providers and/or local government offices between 2011 and 2014. We previously reported that the number of live births registered in JECS accounted for approximately 45% of total live births within the study area [2]. The JECS protocol was reviewed and approved by the Ministry of the Environment’s Institutional Review Board on Epidemiological Studies and the Ethics Committees of all participating institutions. JECS was conducted in accordance with the Helsinki Declaration and other nationally valid regulations and guidelines. Written informed consent was obtained from all participants.

### 2.2. Assessment during Pregnancy

Complete Blood Count and Chemistry Panel

Non-fasting maternal blood samples were collected by medical staff when the pregnant women visited co-operating health care providers during periods named in JECS as MT1 (gestational age 12–16 weeks) and/or MT2 (gestational age 22–28 weeks), respectively. Volumes of 32 mL at MT1 and 33 mL at MT2 of whole blood samples were collected into 5 tubes. The following biomarkers were assayed by a contract clinical laboratory (SRL, Inc., a commercial laboratory in Tokyo, Japan): white blood cell count (WBC), red blood cell count (RBC), hemoglobin (Hb), hematocrit (Ht), mean corpuscular volume (MCV), mean corpuscular hemoglobin (MCH), mean corpuscular hemoglobin concentration (MCHC), platelet count (PLT), HbA1c, total cholesterol (T-Cho), low-density lipoprotein cholesterol (LDL-C), high density lipoprotein cholesterol (HDL-C), free cholesterol (F-Cho), triglycerides (TG), total protein (TP), and albumin (Alb). 

The methods used to measure the biomarkers were as follows: WBC, flow cytometry with semiconductor laser (SYSMEX XE-2100); RBC and PLT, hydrodynamic focusing DC detection (SYSMEX XE-2100); Hb, SLS-Hemoglobin Method (SYSMEX XE-2100); Ht, RBC pulse height detection (SYSMEX XE-2100); MCV, MCH, and MCHC, calculated using RBC, Hb, and Ht values (SYSMEX XE-2100); HbA1c, high-performance liquid chromatography (ADAMS-A1c HA-8160); T-Cho and F-Cho, enzymatic method (HITACHI 7700 SERIES); LDL-C, accelerator selective detergent (HITACHI 7700 SERIES); HDL-C, liquid selective detergent (HITACHI 7700 SERIES); TG, enzymatic method using glycerol phosphate oxidase (HITACHI 7700 SERIES); TP, biuret (HITACHI 7700 SERIES); and Alb, bromocresol green (HITACHI 7700 SERIES).

HbA1c was calculated using reference values from the National Glycohemoglobin Standardization Program or Japan Diabetes Society. For HbA1c values calculated using reference values from the Japan Diabetes Society, the following formula was used: Calculated HbA1c = 1.02 × HbA1c (Japan Diabetes Society) + 0.25 [9,14]. 

WBC, RBC, Hb, Ht, MCV, MCH, MCHC, PLT, HbA1c, LDL-C, and HDL-C were measured only at MT1.

Questionnaires

Self-administered questionnaires were completed by the pregnant women during MT1 and MT2. Self-administered questionnaires covered demographic factors, medical and obstetric history, physical and mental health, lifestyle, occupation, environmental exposure at home and in the workplace, housing conditions, and socioeconomic status [2]. In this study, we extracted data on the following baseline demographic and health characteristics: age, marital status, educational attainment, household income, occupation [15], smoking habit [16], alcohol consumption [16], height, weight, body mass index (BMI), and parity. 

Height, pre-pregnancy weight, and parity were transcribed from medical records before and at birth and from self-administered questionnaires at MT1. Educational attainment and household income were assessed at MT2 only, while the remaining variables were assessed at MT1 and/or MT2. 

### 2.3. Statistical Analysis

**Figure 1:**
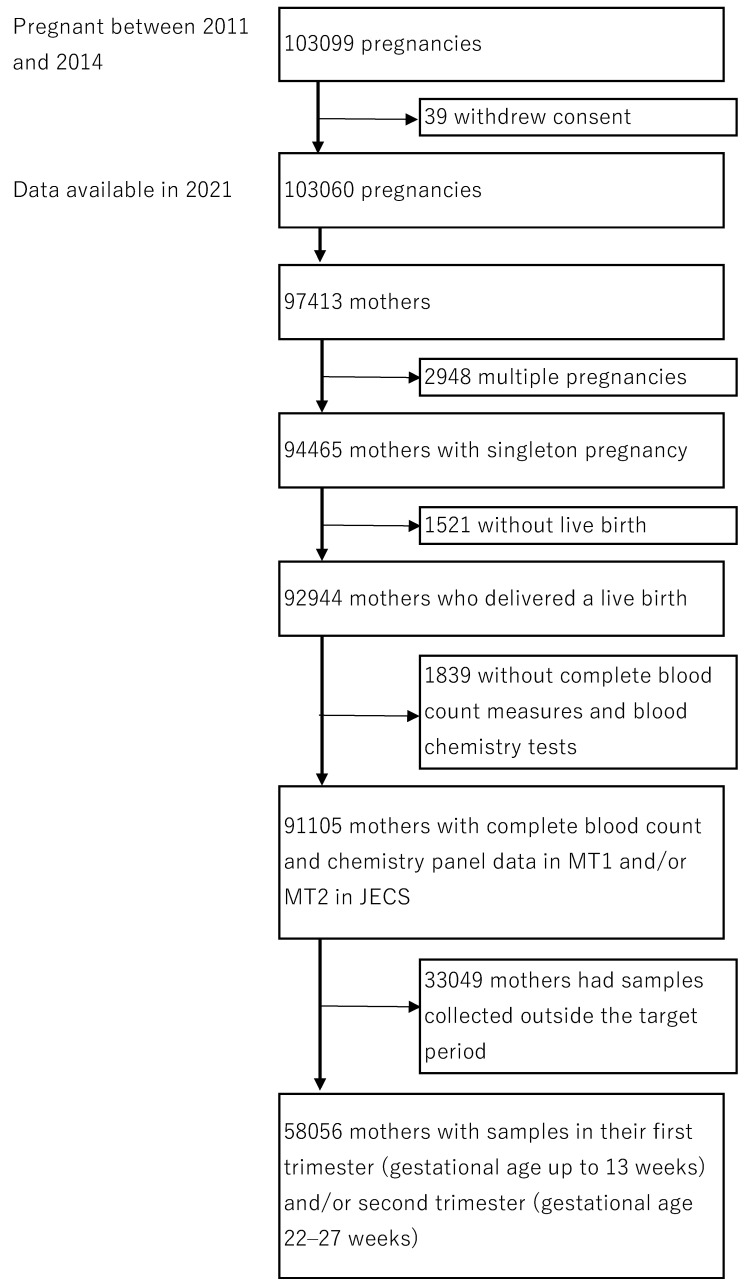
Flow chart of this study.

The present study used the jecs-ta-20190930 dataset, released in October 2019, and modified in June 2021. To be eligible for the study, pregnant women had to have undergone a complete blood count and chemistry panel in the first and/or second trimester and had to have a singleton pregnancy and live birth. Among 103,099 pregnancies, the study excluded women who withdrew consent (*n* = 39). A total of 103,060 pregnancies were identified among 97,413 mothers. We further excluded those with multiple pregnancies (*n* = 2948), without live birth (*n* = 1521), and without complete blood count and chemistry panel data (*n* = 1839). Among the 91,105 pregnant women with complete blood count and chemistry panel data in MT1 and/or MT2, we used the 58,056 samples collected in the first trimester (gestational age up to 13 weeks in MT1: *n*= 23,709) and/or second trimester (gestational age 22–27 weeks in MT2: *n*= 49,857) based on the definition used in clinical settings and previous research. Finally, data for this study were obtained from 58,056 mothers with complete blood count and chemistry panel data in the first trimester and/or second trimester, singleton pregnancy, and live birth (Figure 1). 

First, we summarized a wide range of maternal baseline demographic and health characteristics in the first trimester and/or second trimester. Second, we summarized complete blood count and chemistry panel data in the first trimester and second trimester. These variables were reported as mean (standard deviation: SD) and 10, 25, 50, 75, and 95 percentiles. Third, we summarized complete blood count and chemistry panel data collected in the first trimester for 6 regions in Japan: Hokkaido and Tohoku (Hokkaido, Miyagi, Fukushima), Kanto (Chiba, Kanagawa), Chubu (Koshin, Toyama, Aichi), Kinki (Kyoto, Osaka, Hyogo), Chugoku and Shikoku (Tottori, Kochi), and Kyushu (Fukuoka, and South Kyushu = Okinawa). All descriptive statistics were conducted using SAS version 9.4.

## 3. Results

Female participants in JECS in their first trimester with singleton pregnancies and live births had a mean (SD) age of 30.9 (4.5) years. About 95.7% were married, and 29.7% were homemakers, while 22.0% were professional or engineering workers. In terms of lifestyle habits, 59.7% were never smokers, and 35.3% were never alcohol drinkers. Mean BMI was 21.2 (3.3) kg/m^2^, and 44.7% were primiparas. Among women in their second trimester, 65.0% had graduated from college, junior college, university, or graduate school, and 34.7% and 33.2% had a household income of 2 to <4 million Japanese yen and 4 to <6 million Japanese yen, respectively (Table 1). 

Table 2 shows the women’s complete blood count and chemistry panel profile in the first and second trimester. The mean (SD) values in the first trimester were as follows: WBC, 7866 (1906)/μL; RBC, 417 (33) 10^4^/μL; Hb, 12.4 (1.0) g/dL; Ht, 37.1 (2.6)%; MCV, 89.2 (4.7) fL; MCH, 29.8 (1.9) pg; MCHC, 33.3 (0.9)%; PLT, 24.8 (5.2) 10^4^/μL; HbA1c, 5.26 (0.26)%; T-Cho, 181 (28) mg/dL; LDL-C, 95 (23) mg/dL; HDL-C, 73 (13) mg/dL; F-Cho, 41 (7) mg/dL; TG, 109 (47) mg/dL; TP, 6.9 (0.4) g/dL; and Alb, 4.1 (0.2) g/dL. The mean (SD) values in the second trimester were as follows: T-Cho, 246 (38) mg/dL; F-Cho, 61 (9) mg/dL; TG, 183 (70) mg/dL; TP, 6.5 (0.4) g/dL, and Alb, 3.6 (0.2) g/dL.

When the data were stratified by six regions in Japan (Table 3), the regional difference was a maximum of 5.0% for TG and 4.2% for RBC compared with all participants. PLT, Alb, HDL-C, and TP showed a maximum regional difference of 2.6%, 1.5%, 1.2% and 1.2%, respectively, compared with all participants. The remaining complete blood count and chemistry panel variables showed a regional difference of less than 1%.

## 4. Discussion

In this descriptive study, we confirmed a wide range of maternal baseline demographic and health characteristics for female participants and summarized the mean values of parameters of a complete blood count and chemistry panel conducted during the first trimester and second trimester in pregnant women in JECS, who are regarded as being representative of the general Japanese population [3]. We also examined regional differences in these variables in Japan. 

Although there are currently no standardized complete blood count and chemistry panel data for pregnant women in Japan, the Japan Society of Obstetrics and Gynecology has published a guideline for the first trimester (gestational age up to 13 weeks) [17] for several complete blood count measures. According to this guideline, the present JECS data showed that 97.6%, 97.1%, and 98.6% of pregnant women were in the normal range for WBC (<12,000/μL), Hb (≥10.5 g/dL), and PLT (≥15 × 10^4^/μL), respectively [17]. The Japan Society of Laboratory Medicine has published reference ranges for laboratory test parameters for the Japanese population [18]. The mean values for WBC, RBC, Hb, Ht, MCV, MCH, MCHC, PLT, HbA1c, T-Cho, LDL-C, HDL-C, TG, TP, and Alb for women in their first trimester in this study fell within these reference ranges [18]. We also confirmed that the present data were consistent with those reported in previous studies in JECS [9,19,20].

The mean values for T-Cho, F-Cho, and TG increased between the first trimester and second trimester in this study. A recent study reported that an increase in cholesterol levels is observed during pregnancy and that it is considered a normal adaptive response to the development of the fetus [21]. Maternal cholesterol increases throughout gestation by 50–70% [22], particularly in the second and third trimesters [23]. T-Cho increases up to 39% and TG up to 138% in the third trimester [24]. Although accumulated evidence shows that cholesterol levels are increased during late gestation, the present study revealed that T-Cho, F-Cho, and TG rose 36%, 49%, and 68% between the first and second trimester, respectively. In general, maternal serum Alb levels fall as pregnancy progresses [18], especially during late gestation [25]. The present study showed that Alb decreased by 12% between the first and second trimester, suggesting that Alb levels may begin to gradually decline in early pregnancy. 

Stratified analysis of complete blood count and chemistry panel data by region in Japan showed there was a maximum regional difference of 5.0%. While we speculate that differences in regional cultural lifestyle such as dietary habits within Japan may explain the variability in complete blood count and chemistry panel data, the precise reason is unclear. Additional studies that consider regional characteristics may be needed to establish standard values for complete blood count and chemistry panel parameters for pregnant women. Moreover, future studies in JECS using these variables should consider regional differences when examining the effect of maternal biochemistry on child health and development. Based on the results for a wide range of maternal baseline demographic and health characteristics, future studies that have similar characteristics to this study also should consider regional differences.

## 5. Conclusions

This descriptive study summarized the mean values of parameters of a complete blood count and chemistry panel conducted during the first trimester and second trimester in pregnant women in JECS. These data will be useful for future studies in JECS that aim to examine the effect of maternal biochemistry on child health and development. 

## Figures and Tables

**Table 1:** Baseline demographic and health characteristics of pregnant women in their first and/or second trimester.

Variable	First Trimester (Gestational Age Up to 13 Weeks) *n* = 23,709	Second Trimester (Gestational Age 22–27 Weeks) *n* = 49,857
Age (years)	30.9 (4.5)	31.7 (5.0)
Marital status (%)		
Married	95.7	-
Unmarried	3.6	-
Divorced/widowed	0.8	-
Educational attainment (%)		
Junior high school	-	4.8
High school/technical college	-	30.2
College/junior college/university/graduate school	-	65.0
Household income (%)		
<2 million Japanese yen/year	-	5.8
2 to <4 million Japanese yen/year	-	34.7
4 to <6 million Japanese yen/year	-	33.2
6 to <8 million Japanese yen/year	-	15.9
8 to <10 million Japanese yen/year	-	6.4
≥10 million Japanese yen/year	-	4.1
Occupation (%)		
Administrative and managerial work	0.6	-
Professional and engineering work	22.8	-
Clerical work	17.0	-
Sales work	5.7	-
Service work	14.5	-
Security work	0.3	-
Agriculture, forestry and fishery work	0.5	-
Manufacturing process work	2.6	-
Transport and machine operation workers/Construction and mining work	0.3	-
Carrying, cleaning, packaging, and related work	0.6	-
Homemaker	29.7	-
Other (students, inoccupation, workers not classifiable by occupation)	5.5	-
Smoking habit (%)		
Never smoker	59.7	58.7
Past smoker	36.0	36.8
Current smoker	4.3	4.5
Alcohol consumption (%)		
Never drinker	35.3	33.8
Past drinker	54.9	63.4
Current drinker	9.8	2.8
Height (cm)	158.3 (5.3)	-
Weight (kg)	53.0 (8.8)	-
BMI (kg/m^2^)	21.2 (3.3)	-
Parity (%)	45.6	-
0	44.7	
1	37.7	
≥2	17.6	

Data indicate mean (standard deviation) or %. “-“ indicates data were not collected.

**Table 2:** Baseline complete blood count and chemistry panel profile of pregnant women in their first and/or second trimester.

Variable	First Trimester (Gestational Age Up to 13 Weeks) *n* = 23,709						Second Trimester (Gestational Age 22–27 Weeks) *n* = 49,857					
	Mean (SD)	10%	25%	50%	75%	90%	Mean (SD)	10%	25%	50%	75%	90%
White blood cell count (/μL)	7866 (1906)	5600	6600	7800	9000	10,300	-	-	-	-	-	-
Red blood cell count (10^4^/μL)	417 (33)	375	395	417	439	459	-	-	-	-	-	-
Hemoglobin (g/dL)	12.4 (1.0)	11.2	11.8	12.4	13.0	13.6	-	-	-	-	-	-
Hematocrit (%)	37.1 (2.6)	33.8	35.4	37.1	38.9	40.4	-	-	-	-	-	-
Mean corpuscular volume (fL)	89.2 (4.7)	84.0	87.0	89.6	92.1	94.3	-	-	-	-	-	-
Mean corpuscular hemoglobin (pg)	29.8 (1.9)	27.7	29.0	30.0	30.9	31.7	-	-	-	-	-	-
Mean corpuscular hemoglobin concentration (%)	33.3 (0.9)	32.3	32.9	33.4	33.9	34.4	-	-	-	-	-	-
Platelet count (10^4^/μL)	24.8 (5.2)	18.7	21.3	24.4	27.9	31.4	-	-	-	-	-	-
HbA1c (%)	5.26 (0.26)	4.94	5.1	5.25	5.4	5.55	-	-	-	-	-	-
Total cholesterol (mg/dL)	181 (28)	147	161	179	198	217	246 (38)	200	220	244	269	295
Low density lipoprotein cholesterol (mg/dL)	95 (23)	68	79	93	108	124	-	-	-	-	-	-
High density lipoprotein cholesterol (mg/dL)	73 (13)	57	64	72	81	90	-	-	-	-	-	-
Free cholesterol (mg/dL)	41 (7)	33	37	41	45	50	61 (9)	50	55	61	67	73
Triglycerides (mg/dL)	109 (47)	62	77	99	129	167	183 (70)	111	135	169	213	267
Total protein (g/dL)	6.9 (0.4)	6.4	6.6	6.9	7.1	7.4	6.5 (0.4)	6.0	6.2	6.5	6.7	6.9
Albumin (g/dL)	4.1 (0.2)	3.8	3.9	4.0	4.2	4.4	3.6 (0.2)	3.4	3.5	3.6	3.8	3.9

“-” indicates data were not collected.

**Table 3:** Baseline complete blood count and chemistry panel profile of pregnant women in their first trimester by region in Japan.

Variable	Region in Japan											
	Hokkaido and Tohoku *n* = 5422		Kanto *n* = 2163		Chubu *n* = 5283		Kinki *n* = 5521		Chugoku and Shikoku *n* = 1245		Kyushu *n* = 4075	
	Mean (SD)	Median (IQR)	Mean (SD)	Median (IQR)	Mean (SD)	Median (IQR)	Mean (SD)	Median (IQR)	Mean (SD)	Median (IQR)	Mean (SD)	Median (IQR)
White blood cell count (/μL)	7833 (1926)	7700 (6500, 9000)	8088 (1830)	7900 (6800, 9200)	7737 (1888)	7600 (6500, 8900)	8028 (1928)	7900 (6700, 9200)	7536 (1932)	7500 (6400, 8700)	7841 (1873)	7700 (6600, 9000)
Red blood cell count (10^4^/μL)	418 (34)	417 (395, 439)	418 (33.14)	419 (397, 441)	419 (32.53)	418 (397, 440)	416 (32)	416 (394, 437)	411 (33)	411 (389, 431)	417 (33)	416 (395, 438)
Hemoglobin (g/dL)	12.4 (1.0)	12.4 (11.8, 13.1)	12.5 (1.0)	12.5 (11.9, 13.1)	12.4 (1.0)	12.4 (11.9, 13.0)	12.3 (1.0)	12.4 (11.7, 13.0)	12.3 (0.9)	12.3 (11.7, 12.9)	12.4 (1.0)	12.4 (11.8, 13.0)
Hematocrit (%)	37.0 (2.7)	37.0 (35.3, 38.8)	37.4 (2.6)	37.4 (35.8, 39.2)	37.3 (2.6)	37.3 (35.6, 39.1)	37.1 (2.6)	37.1 (35.4, 38.8)	36.8 (2.6)	36.8 (35.1, 38.5)	37.1 (2.6)	37.0 (35.4, 38.9)
Mean corpuscular volume (fL)	88.8 (4.6)	89.2 (86.7, 91.6)	89.5 (4.3)	89.9 (87.4, 92.2)	89.3 (4.6)	89.7 (87.1, 92.2)	89.3 (4.8)	89.8 (87.1, 92.3)	89.8 (4.3)	90.0 (87.8, 92.5)	89.2 (4.7)	89.7 (86.9, 92.0)
Mean corpuscular hemoglobin (pg)	29.8 (1.9)	30.0 (29.0, 30.9)	29.8 (1.8)	30.0 (29.1, 30.8)	29.8 (1.9)	30.0 (29.0, 30.9)	29.7 (2.0)	30.0 (29.0, 30.9)	30.0 (1. 8)	30.3 (29.2, 31.2)	29.7 (2.0)	30.0 (29.0, 30.9)
Mean corpuscular hemoglobin concentration (%)	33.5 (0.9)	33.5 (33.0, 34.1)	33.3 (0.9)	33.3 (32.8, 33.9)	33.3 (0.9)	33.4 (32.8, 33.9)	33.2 (1.0)	33.3 (32.7, 33.8)	33.4 (0.9)	33.4 (33.0, 34.0)	33.3 (0.9)	33.4 (32.8, 33.9)
Platelet count (10^4^/μL)	25.0 (5.2)	24.6 (21.5, 28.1)	25.1 (5.1)	24.8 (21.6, 28.1)	24.4 (5.2)	24.0 (20.9, 27.5)	25.0 (5.1)	24.6 (21.5, 28.1)	24.2 (4.9)	23.8 (20.8, 27.3)	24.9 (5.2)	24.6 (21.3, 28.0)
HbA1c (%)	5.26 (0.28)	5.25 (5.10, 5.40)	5.26 (0.25)	5.25 (5.10, 5.40)	5.27 (0.26)	5.25 (5.10, 5.45)	5.25 (0.25)	5.25 (5.10, 5.40)	5.24 (0.24)	5.25 (5.10, 5.40)	5.26 (0.27)	5.25 (5.10, 5.40)
Total cholesterol (mg/dL)	180 (29)	178 (160, 197)	182(29)	180 (162, 199)	180 (28)	178 (160, 197)	182 (29)	180 (162, 199)	181 (26)	179 (163, 198)	179 (28)	177 (160, 196)
Low density lipoprotein cholesterol (mg/dL)	95 (24)	93 (79, 109)	95 (24)	93 (79, 110)	94 (23)	92 (78, 108)	96 (23)	94 (80, 109)	95 (22)	93 (80, 109)	94 (23)	91 (78, 108)
High density lipoprotein cholesterol (mg/dL)	72 (13)	71 (63, 80)	74 (13)	73 (65, 82)	73 (13)	72 (64, 81)	74 (13)	73 (65, 82)	73 (13)	72 (65, 81)	72 (13)	72 (63, 80)
Free cholesterol (mg/dL)	41 (7)	41 (36, 45)	41 (7)	41 (37, 46)	41 (7)	41 (36, 45)	42 (7)	41 (37, 46)	42 (6)	41 (37, 46)	41 (7)	41 (37, 45)
Triglycerides (mg/dL)	107 (47)	98 (76, 127)	109 (48)	100 (77, 131)	108 (46)	98 (76, 128)	110 (48)	100 (77, 131)	115 (47)	104 (82, 135)	110 (46)	100 (78, 131)
Total protein (g/dL)	6.9 (0.4)	6.9 (6.6, 7.1)	6.9 (0.4)	6.9 (6.6, 7.1)	6.9 (0.4)	6.9 (6.7, 7.2)	6.9 (0.4)	6.9 (6.6, 7.2)	6.8 (0.4)	6.8 (6.6, 7.1)	6.9 (0.4)	6.9 (6.6, 7.1)
Albumin (g/dL)	4.1 (0.3)	4.0 (3.9, 4.2)	4.1 (0.3)	4.0 (3.9, 4.2)	4.1 (0.2)	4.1 (3.9, 4.2)	4.1 (0.2)	4.1 (3.9, 4.2)	4.0 (0.2)	4.0 (3.8, 4.1)	4.0 (0.2)	4.0 (3.9, 4.2)

Data indicate mean (standard deviation) and median (IQR).

## Data Availability

Data are unsuitable for public deposition due to ethical restrictions and the legal framework of Japan. It is prohibited by the Act on the Protection of Personal Information (Act No. 57 of 30 May 2003, amendment on 9 September 2015) to publicly deposit the data containing personal information. Ethical Guidelines for Medical and Health Research Involving Human Subjects enforced by the Japan Ministry of Education, Culture, Sports, Science and Technology and the Ministry of Health, Labour and Welfare also restrict the open sharing of the epidemiologic data. All inquiries about access to data should be sent to: jecs-en@nies.go.jp. The person responsible for handling enquiries sent to this e-mail address is Shoji F. Nakayama, JECS Programme Office, National Institute for Environmental Studies.
